# Cafeteria Diet Feeding in Young Rats Leads to Hepatic Steatosis and Increased Gluconeogenesis under Fatty Acids and Glucagon Influence

**DOI:** 10.3390/nu10111571

**Published:** 2018-10-23

**Authors:** Antonio Sueiti Maeda Júnior, Jorgete Constantin, Karina Sayuri Utsunomiya, Eduardo Hideo Gilglioni, Fabiana Rodrigues Silva Gasparin, Fernando Olinto Carreño, Solange Marta Franzói de Moraes, Márcio Rocha, Maria Raquel Marçal Natali, Cristiane Vizioli de Castro Ghizoni, Adelar Bracht, Emy Luiza Ishii-Iwamoto, Rodrigo Polimeni Constantin

**Affiliations:** 1 Department of Biochemistry, Laboratory of Biological Oxidations and Laboratory of Experimental Steatosis, State University of Maringá, Maringá 87020-900, Paraná, Brazil; jrmaedaopcao@gmail.com (A.S.M.J.); jconstantin@uem.br (J.C.); ksutsunomiya2@uem.br (K.S.U.); gilglioni@hotmail.com (E.H.G.); fabygasparin@hotmail.com (F.R.S.G.); fer.carreno@gmail.com (F.O.C.); marciorocha@mail.com (M.R.); eliiwamoto@uem.br (E.L.I.-I.); 2 Department of Morphophysiological Sciences, State University of Maringá, Maringá 87020-900, Paraná, Brazil; smfmoraes@uem.br (S.M.F.d.M.); mrmnatali@uem.br (M.R.M.N.); 3 Department of Biochemistry, Laboratory of Liver Metabolism, State University of Maringá, Maringá 87020-900, Paraná, Brazil; crisvizioli@gmail.com (C.V.d.C.G.); adebracht@uol.com.br (A.B.)

**Keywords:** obesity, high caloric density food, NAFLD, glucose production, hyperglycemia, hemodynamic changes

## Abstract

Gluconeogenesis overstimulation due to hepatic insulin resistance is the best-known mechanism behind elevated glycemia in obese subjects with hepatic steatosis. This suggests that glucose production in fatty livers may differ from that of healthy livers, also in response to other gluconeogenic determinant factors, such as the type of substrate and modulators. Thus, the aim of this study was to investigate the effects of these factors on hepatic gluconeogenesis in cafeteria diet-induced obese adult rats submitted to a cafeteria diet at a young age. The livers of the cafeteria group exhibited higher gluconeogenesis rates when glycerol was the substrate, but lower rates were found when lactate and pyruvate were the substrates. Stearate or glucagon caused higher stimulations in gluconeogenesis in cafeteria group livers, irrespective of the gluconeogenic substrates. An increased mitochondrial NADH/NAD^+^ ratio and a reduced rate of ^14^CO_2_ production from [^14^C] fatty acids suggested restriction of the citric acid cycle. The higher glycogen and lipid levels were possibly the cause for the reduced cellular and vascular spaces found in cafeteria group livers, likely contributing to oxygen consumption restriction. In conclusion, specific substrates and gluconeogenic modulators contribute to a higher stimulation of gluconeogenesis in livers from the cafeteria group.

## 1. Introduction

The prevalence of childhood obesity worldwide is increasing to the point of being considered a public health concern [1,2] and is attributed to the ingestion of the Western diet, rich in industrialized palatable high caloric density food [3,4]. The consequences of such diet during childhood can last for a lifetime. It is estimated that obese children or adolescents are five times more susceptible to be obese in adulthood than those who are not obese [5]. The excess of adipose tissue is frequently associated with other metabolic comorbidities, including non-alcoholic fatty liver disease (NAFLD), the most common liver illness among children and adolescents in industrialized countries [6]. NAFLD usually occurs in association with other cardio-metabolic disorders of the metabolic syndrome [7,8], a group of clinic-laboratorial features that is believed to be initiated by the expansion of the adipose visceral tissues, leading to liver lipid accumulation, atherogenic dyslipidemia, hypertension, and chronic hyperglycemia [7,9]. The linkage between NAFLD and chronic hyperglycemia involves an impaired capacity of insulin to suppress gluconeogenesis in fatty livers, resulting in high hepatic glucose synthesis and increased pancreatic insulin secretion [10,11]. This observation reveals a paradox in hepatic insulin resistance since some metabolic pathways appear to remain sensitive to insulin regulation, as *de novo* lipogenesis, while others become resistant, as gluconeogenesis [12,13,14].

When gluconeogenesis is favoured by the glucagon/insulin ratio in the portal vein, the glucose production rate is mainly determined by the substrates provided by other tissues [15]. In obese subjects, it was shown that high muscular protein catabolism with a subsequent influx of amino acids to the liver increases gluconeogenic rates [16]. More recently, our research group demonstrated that the hepatic capacity to produce glucose from glutamine and alanine is lower in fatty livers from cafeteria diet-fed rats with obesity compared to the control condition [17]. In the current work, we have hypothesized that besides responding differently to the substrate type as well as to insulin, fatty livers must also have disturbed responses to the positive gluconeogenic modulators glucagon and fatty acids, both usually found in high levels in the blood of obese subjects with NAFLD [18,19,20,21]. To test this hypothesis, we assessed the gluconeogenic rates under the influence of glucagon or fatty acids in perfused livers from rats fed a standard diet and fatty livers from obese rats fed a cafeteria diet. Because we were interested in studying metabolic dysfunctions in animals that gain excessive body weight and develop fatty liver by voluntary hyperphagia at a younger age, the rats were fed a cafeteria diet starting at weaning. The results provide an essential step forward in understanding the interaction between obesity and NAFLD development at early stages of life and the increased hepatic glucose production underlying chronic hyperglycemia commonly observed in the adult phase.

## 2. Materials and Methods

### 2.1. Materials

The liver perfusion apparatus and the rapid sampling apparatus for multiple-indicator dilution (MID) experiments were built in the workshops of the University of Maringá. Enzymes and coenzymes were purchased from the Sigma Chemical Company (St. Louis, MO, USA) as reagent-grade chemicals, salts, buffers, and substrates. [1-^14^C]octanoic acid (25 mCi/mmol) was purchased from New England Nuclear (Boston, MA, USA). [1-^14^C]stearic acid (50 mCi/mmol), [^3^H]water (20 μCi/mmol), and the biodegradable counting scintillant solution (BCS^®^) were purchased from Amersham Pharmacia Biotech (Buckinghamshire, UK). [U-^14^C]sucrose (435 mCi/mmol) was purchased from Perkin-Elmer (Boston, MA, USA). All other reagents were obtained from the highest available grade.

### 2.2. Animals

Weaned male 21-day-old Wistar rats (*Rattus novergicus*) were provided from the Animal Facility of the University of Maringá. Animals were kept in polypropylene cages (3 animals/box) in a light-dark cycle of 12 h at 22–24 °C. Rats were arbitrarily divided into the following 2 groups: control (CON) and cafeteria diet-induced obese (CAF) rats. The CON group was fed *ad libitum* with rodent standard diet, while the CAF group was fed Brazilian industrial food (cafeteria diet) for 14 weeks. Body weights were assessed weekly. All experiments were conducted in strict adherence to the guidelines of the Ethics Committee for Animal Experimentation of the University of Maringá (protocol 113/2010) in accordance with the internationally accepted recommendation for the care and use of animals.

### 2.3. Diet

The CON group had free access to water and standard rodent chow (Nuvilab-Nuvital^®^, Colombo, Brazil), recommended by the National Research Council and the National Institutes of Health (Bethesda, MD, USA). This standard rodent chow is composed of whole ground corn, soybean meal, and wheat bran and is supplemented with minerals, amino acids, and vitamins, totalling 16.15 kJ/g (64% carbohydrate, 26% protein, and 10% lipid). The CAF group had free access to Brazilian industrialized and high-density caloric food, such as cheese- or bacon-flavoured chips, marshmallows, peanut candy, filled and wafer cookies, sausage, mortadella, and soda, which were all offered in excess [22,23]. The energy density of the cafeteria diet (taken from the daily offering of each component) totalled 16.28 kJ/g on average (73% carbohydrate, 10% protein, and 17% lipid). The energy density from carbohydrate, protein, and lipid in CON and CAF rats was calculated in accordance with nutritional information provided by the manufacturers. The sources of the main macronutrients were variable (type and proportion) in each food of the cafeteria diet and were provided by the following: corn and wheat flour, textured soy protein, poultry and pork meat, pork skin and giblets (liver, kidneys, heart), poultry fats, hydrogenated vegetable fat, corn and cassava starch, whey powder, gelatin, roasted peanut, sugar, glucose syrup, invert sugar, and/or corn cream. Animals also had free access to standard diet and water. Both the standard and cafeteria diet were replaced daily with fresh food. Food ingestion (g and kJ) was evaluated by weighing each constituent of the offered diet and corresponding leftovers on the succeeding day. The data was used for the calculation of the cumulative energy intake during the whole experimental period.

### 2.4. Characterization of Animals

From the first day, all animals were weighed weekly during the dietary treatment period. At the end of treatment, retroperitoneal, mesenteric, periepididymal, and interscapular fat deposits were weighed, and the adiposity index was calculated from the sum of these tissue weights and expressed in g per 100 g body weight. For comparative purposes, freshly isolated livers of CON and CAF rats were also weighed after the treatment.

### 2.5. Hepatic Glycogen Measurement

Glycogen content was quantified in freshly isolated livers of fed and overnight (12 h) fasted rats at the end of dietary treatment. Rats were anesthetized with a mixture of sodium thiopental-lidocaine injection (50–4 mg/kg, intraperitoneally), and approximately 2 g of liver was rapidly removed and freeze-clamped in liquid nitrogen. These samples were homogenized and extracted using 10 mL of 6% HClO_4_. The supernatant was neutralized with 5 M K_2_CO_3_ and used for the enzymatic glycogen assay [24], and the amount of glycogen was expressed as µmol glucosyl units × g liver^−1^.

### 2.6. Determination of Hepatic Total Lipid and Protein Contents

The hepatic total lipid and protein contents were measured at the end of treatment. Fed rats were anesthetized with a mixture of sodium thiopental-lidocaine injection (50–4 mg/kg, intraperitoneally). After the surgical procedure, CON and CAF rat livers were first perfused in a non-recirculating mode for approximately 2 min with Krebs/Henseleit-bicarbonate buffer (pH 7.4, without bovine serum albumin) to remove blood from the organ (exsanguination). Shortly thereafter, the livers were excised, weighed, freeze-clamped in liquid nitrogen, crushed in a liquid nitrogen-cooled mortar, and lyophilized for 24 h. The final content was transferred to 50 mL Falcon^®^ conical centrifuge tubes and stored at −80 °C until determination. Total nitrogen (TN) was determined using the Tecnal TE-0036/1 apparatus (Tecnal, Piracicaba, São Paulo, Brazil) following method n° 990.03 of the AOAC (1990), and crude protein (CP) was estimated as TN × 6.25 [25]. The hepatic total protein content was expressed as mg protein × g liver wet weight^−1^. The determination of total lipid content was carried out by applying the method n° 7060 of the AOAC (1990). This method utilizes ether for lipid extraction since it was found to be an excellent lipid extractant [25]. The ether extract was assessed using a Tecnal TE-044/1 apparatus (Tecnal, Piracicaba, São Paulo, Brazil). The hepatic total lipid content was expressed as mg lipid × g liver wet weight^−1^.

### 2.7. Liver Histochemical Analysis

For histochemical analysis, fed rats were euthanized with a mixture of sodium thiopental-lidocaine injection (150–4 mg/kg, intraperitoneally), and liver samples were rapidly removed, frozen in liquid nitrogen, stored at −80 °C, and cut into 10-µm-thick semi-serial histological sections using the Leica^®^ CM1850 cryostat (Leica Biosystems, Wetzlar, Germany). Sudan III staining was prepared according to a standard method [26] for histochemical identification of lipid vesicles. Images were captured with a 20× objective (100 images/group, 25 images/animal) of an optical microscope (Olympus BX41^®^, Tokyo, Japan) with a QColor3^®^ camera (Olympus American INC, Surrey, BC, Canada), coupled to the software Q-Capture^®^. Centrilobular veins in the centre of the images were used as criteria in the choice of fields to be captured. Image Pro-Plus^®^ 4.5 software was used to determine the area occupied by lipid inclusions in each image, which is related to the total area of the image by calculating the area of lipid inclusions in µm × 100^−1^ × total area of the image^−1^.

### 2.8. Liver Perfusion Experiments

The experiments were performed at the end of treatment (14 weeks). For the surgical procedure, animals (CON and CAF rats) were anesthetized with a mixture of sodium thiopental-lidocaine injection (50–4 mg/kg, intraperitoneally). To perform the hemoglobin-free, non-recirculating perfusion [27], the livers of overnight (12 h) fasted rats were used in all perfusion experiments. After cannulating the portal and cava veins, the liver was positioned in a plexiglass chamber. A constant flow was maintained by a peristaltic pump (Minipuls 3, Gilson, France) and adjusted to 34–36 mL/min, depending on liver weight. The perfusion fluid was the fatty acid (FA)-free bovine serum albumin Krebs/Henseleit-bicarbonate buffer (pH 7.4) saturated with a mixture of oxygen and carbon dioxide (95:5) using a membrane oxygenator with simultaneous temperature adjustment to 37 °C. The Krebs/Henseleit-bicarbonate buffer composition was as follows: 115 mM NaCl, 25 mM NaHCO_3_, 5.8 mM KCl, 1.2 mM Na_2_SO_4_, 1.18 mM MgCl_2_, 1.2 mM NaH_2_PO_4_, and 2.5 mM CaCl_2_. Substrates were added to the perfusion fluid according to the experimental protocol. In some experiments, 2 mM lactate + 0.2 mM pyruvate were infused in the absence and presence of a stearate (0.2 mM) + [1-^14^C]stearate (50 mCi/mmol) mixture in sodium salt form complexed with FA-free bovine serum albumin (0.15 mM). In other experiments, the octanoate (0.3 mM) + [1-^14^C]octanoate (25 mCi/mmol) mixture was infused as sodium salt complexed with FA-free bovine serum albumin (0.00375 mM). The use of [1-^14^C]-labelled FAs is effective at measuring the citric acid cycle (CAC) flux via acetyl-CoA labelling [28]. Consequently, ^14^CO_2_ production can be regarded as a CAC activity indicator. The following exogenous substrates were also infused and solubilized in Krebs/Henseleit-bicarbonate perfusion fluid (pH 7.4) containing 3.74 μM bovine-serum albumin: 2.0 mM glycerol or 2.0 mM glycerol + 10 nM glucagon, 2.0 mM lactate, or 2.0 mM lactate + 10 nM glucagon. At the end of the perfusion experiments, the livers were removed and weighted to allow precise metabolic calculation.

### 2.9. Analytical Assays of Liver Perfusion Experiments

After stabilization of oxygen consumption, experiments were initiated and the effluent fluid samples were collected at 2 min intervals and analysed for their metabolic contents. Glucose, lactate, pyruvate, acetoacetate, and β-hydroxybutyrate were assayed by means of standard enzymatic procedures [24], The oxygen concentration in the outflowing perfusate was continuously monitored by a teflon-shielded platinum electrode adequately positioned in a plexiglass chamber at the perfusate output [27,29]. The carbon dioxide production from [1-^14^C]octanoate or [1-^14^C]stearate was measured by trapping ^14^CO_2_ in phenylethylamine [30]. Radioactivity was measured by liquid scintillation spectroscopy. The following scintillation solution was used: toluene/ethanol (2/1) containing 5 g/L 2,5-diphenyloxazole and 0.15 g/L 2,2-p-phenylenebis(5-phenyloxazole). The metabolic rates were calculated from the input and output difference and the total flow rate and analysed in reference to the liver wet weight. All metabolic fluxes in CON and CAF rat livers were expressed as µmol × (min × g wet liver)^−1^.

The time-course change of metabolite fluxes in the experimental series allowed for the calculation of the area under the curve (AUC) for each parameter (except for the β-hydroxybutyrate to acetoacetate ratio, which is not properly classified as a metabolite). This procedure allowed for the discrimination of the total metabolite amount that was derived from endogenous or exogenous sources by subtracting the basal metabolite production (before substrate infusion). It was also possible to distinguish the metabolite production derived from administration of each exogenous substrate. In specific occasions, when metabolic fluxes were above basal production (absence of exogenous gluconeogenic substrates), the area would be the same as the definite integral, in which initial and final times (*t_i_* and *t*, respectively) are the independent variables, as follows:

(1) $$Area=\int_{t_{i}}^{t}f(t).dt$$

However, when the fluxes are below the basal production, the definite integral is negative, and the area is then given by the following:

(2) $$Area=-\int_{t_{i}}^{t}f(t).dt$$

### 2.10. Multiple-Indicator Dilution Experiments

Multiple-indicator dilution (MID) experiments were conducted using the single injection method [31]. The experiments were performed by injecting 80 μL of a mixture containing 5 μCi [^3^H]water and 1.5 μCi [^14^C]sucrose into the portal vein after oxygen consumption stabilization. The effluent perfusate was collected in 0.5- to 2.0-s fractions over a period of 90 s following injection by means of a specially designed fraction collector. The samples were added to a biodegradable counting scintillant solution (BCS^®^) to measure radioactivity by liquid scintillation, with isotope discrimination for the simultaneous quantification of ^3^H and ^14^C present in the samples. [^14^C]sucrose was counted after [^3^H]water elimination by freeze-drying, and the latter was computed from the difference between total [^14^C]sucrose and [^3^H]water. All dilution curves were normalized as the amount in the effluent sample × (s × total amount injected)^−1^. Livers from CON and CAF rats in the following two different metabolic conditions were used: *ad libitum* fed rats and overnight (12 h) fasted rats. The hepatic glycogen levels from the fed condition were elevated, whereas for the overnight fasting (12 h) condition the glycogen levels were very low [32].

### 2.11. Calculation of Multiple-Indicator Dilution Experiments

The transit time in the large vessels (*t*_0_) and the ratio of intracellular to extracellular water spaces (*θ*) were derived from an optimized superposition of the normalized outflow profile of [^3^H]water (*Q_water_*(*t*)) on the outflow profile of [^14^C]sucrose (*Q_suc_*(*t*)), as predicted by the Goresky’s model [33]. The superposition of the outflow profiles can be described as the following:

(3) $$Q_{water}(t)={\left[\frac{1}{1+\theta }\right]}^{}Q_{suc}\left(\frac{t-t_{0}}{1+\theta }\right)+t_{0}$$

*Q_water_*(*t*) is the impulse response of the liver to [^3^H]water and *Q_suc_* ([*t – t*_0_]/[1 + *θ*] + *t*_0_) is the [^14^C]sucrose curve at time ([*t – t*_0_]/[1 + *θ*] + *t*_0_). Equation (3) assumes a flow-limited distribution of both tracers. Optimization of the superposition was accomplished by means of a non-linear iterative least-squares procedure. Integrals and derivatives of the experimental [^3^H]water and [^14^C]sucrose curves were calculated analytically after approximating the curves by cubic splines, with monoexponential extrapolation to infinite time from the last experimental point (90 s). Interpolation between experimental points was achieved by means of a spline-function [34].

The mean transit time of tracers $\left(\bar{t}\right)$ was calculated by means of the trapezoid rule with monoexponential extrapolation to infinity [35], where *Q*(*t*) represents the normalized outflow profile of tracer and *t* represents time after injection, as follows:

(4) $$\bar{t}=\int_{0}^{\infty }Q(t).t^{}dt$$

### 2.12. Mitochondria Isolation

After the 14-week treatment, CON and CAF rat livers were removed and cut into small pieces. These fragments were suspended in a medium containing 0.2 M mannitol, 75 mM sucrose, 1.0 mM Tris-HCl (pH 7.4), 1.0 mM EGTA, 0.1 mM PMSF, and 50 mg% (*w*/*v*) FA-free bovine serum albumin. Homogenization was carried out in the same medium by means of a Dounce homogenizer. Thereafter, mitochondria were isolated by differential centrifugation [36] and suspended in the same medium (70–80 mg protein/mL), which was kept at 0–4 °C.

### 2.13. Determination of Oxygen Consumption by Isolated Mitochondria Oxidizing Fatty Acids

Oxygen consumption by isolated mitochondria oxidizing FAs was measured polarographically using a teflon-shielded platinum electrode [29,30,36]. The incubation medium contained 2.0 mM KH_2_PO_4_, 10 mM HEPES (pH 7.2), 0.1 mM EGTA, 130 mM KCl, 5.0 mM MgCl_2_, 0.1 mM 2,4-dinitrophenol, 2.5 mM l-malate, and 50 mg% (*w*/*v*) FA-free bovine serum albumin. Mitochondria (1.0 mg protein/mL) were incubated in final volumes of 2.0 mL. The reaction was initiated by addition of (a) 20 μM palmitoyl-l-carnitine, (b) 20 μM palmitoyl-CoA + 2.0 mM l-carnitine, (c) 20 μM octanoyl-l-carnitine, or (d) 20 μM octanoyl-CoA + 2.0 mM l-carnitine. In this experimental series, oxygen consumption was followed for nearly 6 min, and the rates of oxygen consumption were computed from the slopes of the recorder tracings and expressed as nmol × (min × mg protein)^−1^. Protein content of the mitochondrial suspension was measured by means of Folin and Ciocalteu’s phenol reagent [37] using bovine serum albumin as a standard.

### 2.14. Statistical Analysis

The data in the figures and tables is presented as the mean ± the standard error (SEM). The statistical significance of the differences between parameters obtained in the experiments was evaluated by means of Student’s *t*-test or by two-way analysis of variance (ANOVA) followed by the Bonferroni *posthoc* test, according to the context. The results are discussed in the text using *p* values where *p* < 0.05 was the criterion used for significance. Statistical analysis and the calculation of AUC were performed using GraphPad Software (San Diego, CA, USA).

## 3. Results

### 3.1. Biometrical Parameters, Hepatic Glycogen Measurements, and Determination of Hepatic Total Lipid and Protein Contents in Rats Fed a Standard or a Cafeteria Diet

**Figure 1:**
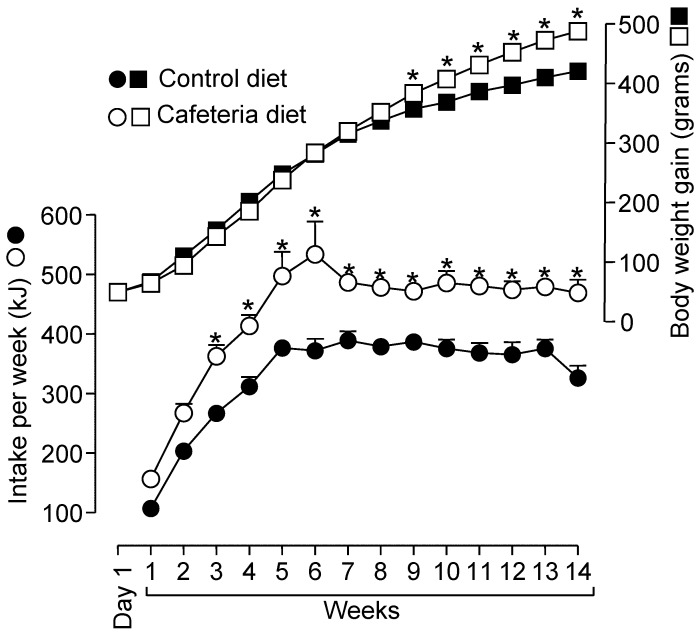
Energy intake in relation to body-weight changes in control and cafeteria-fed rats. Energy intake (kJ) per week (circles) was calculated based on daily food intake and nominal joule content of each individual item in the offered diet. Each experimental point is the mean ± standard error of 21 experiments. Data were first analysed using two-way analysis of variance followed by Bonferroni *post hoc* test for each presented experimental point. *Differences were considered significant at *p* < 0.05.

To follow the development of the cafeteria diet-induced obesity we have measured the food consumption and the body weight gain throughout the entire experimental period and the results are shown in Figure 1. The body weight gain of animals during the 14-week treatment increased linearly until the 8th week without significant difference between CON and CAF groups. After this time, the body weight of CAF rats was higher than of CON rats (*p* < 0.05). As summarized in Figure 1 and Table 1, at the end of the experimental period (14th week), CAF animals presented 16.6% higher body weights than CON animals (*p* < 0.05). The body weight gain over this period was 18.9% (*p* < 0.05) higher in CAF rats compared to CON rats. Both groups ate the same amount of food (by weight) during the entire treatment period (data not shown). However, the total energy ingested was not similar, as shown by the curves in Figure 1. From the 3rd week onwards, CAF animals had a greater energy intake than CON animals (*p* < 0.05).

The body fat content was measured to confirm that the higher body weight gain was due to body fat accumulation (Table 1). CAF rats exhibited a significantly higher adiposity index (+101.3%, *p* < 0.05) than CON rats. In CAF rats, the weight of all fat deposits was substantially increased. Epididymal, retroperitoneal, mesenteric, and interscapular fat deposit weights of CAF animals increased 63.7%, 94.4%, 123.3%, and 83.3%, respectively, compared to CON rats (*p* < 0.05). The weight of CAF rat livers was also higher (+18.1%, *p* < 0.05).

The main hepatic feature of the cafeteria diet-induced obesity, the hepatic steatosis, was confirmed by biochemical measurements of the total liver lipid content. As expected, the liver lipid content was increased in CAF rats (Table 1), whereas the CON group showed a normal lipid content of approximately 1.9% (by weight) [38], this was 5.9% of the liver weight (*p* < 0.05) in CAF animals, a classic steatosis marker. No difference was found between CON and CAF rats regarding the hepatic total protein content. CAF animals also presented higher levels of hepatic glycogen. In the *ad libitum*-fed condition, the levels were 84.0% higher in the CAF group compared to CON group (*p* < 0.05). The relative difference was more pronounced in overnight (12 h) fasted rats. In this condition, the residual hepatic glycogen level in CAF was 113.7% higher than in CON animals (*p* < 0.05).

### 3.2. Liver Histochemical Analysis in Rats Fed a Standard or a Cafeteria Diet

**Figure 2:**
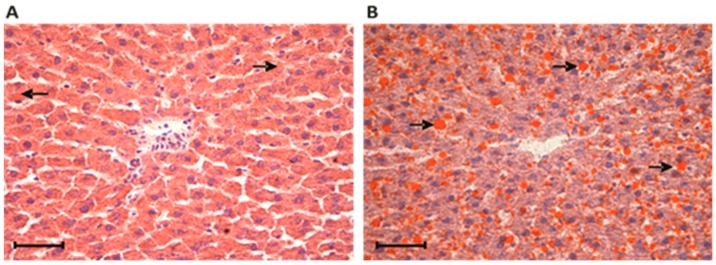
Histochemical analysis of the liver by staining with Sudan III. Frozen sections from the livers of control (**A**) and cafeteria-fed (**B**) rats reveal orange-stained fat-storing cells disposed along the hepatic sinusoids. In the center of the image are centrilobular veins. The area percentage occupied by lipid inclusions (in orange, indicated by arrows) in the livers was significantly lower in the control group (2.34 ± 0.73) compared with the cafeteria group (11.71 ± 2.04). *p* < 0.05, according to Student’s *t* test. Each value is the mean ± SEM of four independent animals. Scale bar 5 μm.

The accumulation of fat in the CAF rat livers was further confirmed by histochemical analyses using the fat-soluble dye Sudan III, as shown in Figure 2. The percentage occupied by lipid inclusions (in orange) in hepatocytes from CON livers was significantly lower (2.3%) compared with that of the CAF group (11.7%, *p* < 0.05).

### 3.3. Metabolic Fluxes Due to Lactate Plus Pyruvate Infusion in the Absence and Presence of Stearate in Livers of Rats Fed a Standard or a Cafeteria Diet

**Figure 3:**
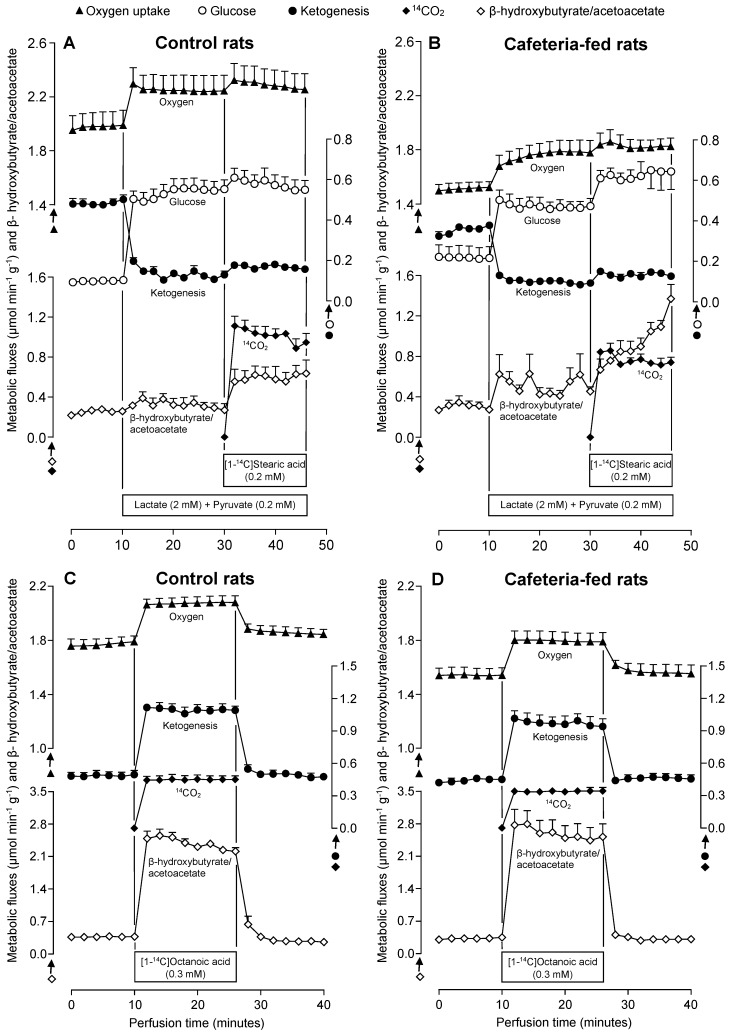
Time courses of the changes in glucose, CO_2_ and ketone bodies production, the ratio between β-hydroxybutyrate and acetoacetate and oxygen consumption in the absence and presence of exogenously added substrates in livers of control (**A**,**C**) and cafeteria-fed (**B**,**D**) rats. Livers of overnight (12-h) fasted rats were perfused as described in Materials and methods. Exogenous substrates were infused at the time intervals indicated by horizontal bars. Each experimental point is the mean ± standard error of 4–5 experiments.

The first set of perfusion experiments was planned to evaluate the gluconeogenic capacity of the livers in both the absence and presence of exogenously added stearate. The experiments were performed with perfused livers of CON and CAF rats previously submitted to overnight (12 h) fasting. After stabilization of oxygen consumption (zero in the time scale of Figure 3A,B), collection of samples was initiated. From 0–10 min, the livers were perfused without exogenous substrate. In the series shown in Figure 3A,B, 2 mM lactate + 0.2 mM pyruvate were infused from 10–46 min. From 30–46 min, 0.2 mM stearate and trace amounts of [1-^14^C]stearate (50 mCi/mmol) were also infused. Oxygen consumption was monitored continuously, and samples of the liquid effluent were analysed for glucose, acetoacetate, β-hydroxybutyrate, and CO_2_ production (30–46 min). Glucose release before gluconeogenic substrate infusion was low in both CON and CAF rat livers, although relatively higher values were found in CAF rats. Glucose production in both groups increased rapidly upon 2 mM lactate + 0.2 mM pyruvate infusion, reaching new steady-state values after approximately 20 min. Under this condition, the rate in CAF rats was smaller compared to the CON group. The simultaneous 0.2 mM stearate infusion in CON livers caused a slight and transient increase in glucose production, but a substantial and sustained stimulation was observed in CAF livers.

Oxygen consumption in the absence of the exogenously added substrate was significantly lower in CAF livers. Gluconeogenic substrate infusion stimulated oxygen consumption in both groups. The stimulation was more pronounced in CON livers compared to CAF livers. The subsequent 0.2 mM stearate infusion induced a small and similar additional increment in oxygen consumption in both groups. The ^14^CO_2_ production from [1-^14^C]stearate, an indicator of CAC activity, was clearly smaller in CAF livers.

The total ketone body production (sum of the acetoacetate and β-hydroxybutyrate production) and the ratio between β-hydroxybutyrate and acetoacetate are also represented in Figure 3A,B. During the 10 min pre-perfusion period in the absence of exogenous substrates, ketogenesis was lower in CAF livers than in perfused CON livers. The 2 mM lactate + 0.2 mM pyruvate infusion at 10 min caused an immediate decrease in total ketone body production. This response was less pronounced in CAF when compared to CON livers so that the difference in ketogenesis found before the substrate infusion was abolished. The concomitant 0.2 mM stearate infusion was not able to restore the total ketone body production in both groups. The β-hydroxybutyrate to acetoacetate ratio until the 30 min perfusion time was not significantly different between the two experimental groups, although the CAF values tended to be higher than those of CON livers. A pronounced difference appeared only when 0.2 mM stearate was infused. In CON livers this ratio increased to a new steady state approximately 4 min after the 0.2 mM stearate infusion; however, there was a progressive increase in CAF livers during the whole period. At the end (46 min perfusion time), the β-hydroxybutyrate to acetoacetate ratio was substantially higher in CAF (1.37 ± 0.14) compared to CON (0.63 ± 0.13) livers (*p* < 0.05).

To facilitate the comparison between experimental series, the AUC for each parameter was calculated and summarized in Table 2. In this case, metabolite production quantified during the 0.2 mM stearate infusion was subtracted from the metabolite amount measured in the presence of gluconeogenic substrates, 2.0 mM lactate + 0.2 mM pyruvate. This data revealed that in the absence of exogenous substrates (in the first 10 min time course in Figure 3A,B), the released glucose in CAF tended to be higher compared to CON livers. On the contrary, the total ketone body production was smaller (−16.5%, *p* < 0.05). Oxygen consumption was also lower in CAF livers (−18.3%, *p* < 0.05). The total glucose amount produced from 2 mM lactate + 0.2 mM pyruvate was lower in CAF compared to CON livers (−41.5%, *p* < 0.05). In this condition (activated gluconeogenesis), the ketone body production was suppressed in both CON and CAF livers. This suppression was more pronounced in the CON group, as denoted by the negative values shown in Table 2 (*p* < 0.05). Under the gluconeogenic condition, the increase in oxygen consumption was similar in both groups. The additional 0.2 mM stearate infusion increased all measured metabolites. The most pronounced effect was in glucose production, which was 4.4-fold higher in CAF than in CON livers (*p* < 0.05). Ketone body production and oxygen consumption were not significantly different between the groups. The ^14^CO_2_ production from [1-^14^C]stearate catabolism was 24.8% lower in CAF compared to CON livers (*p* < 0.05).

### 3.4. Metabolic Fluxes Due to Octanoate Infusion in Livers of Rats Fed a Standard or a Cafeteria Diet

Since some parameters related to FA β-oxidation were altered during the concomitant infusion of stearate, lactate, and pyruvate, experimental series were performed with exogenous octanoate in the absence of gluconeogenic substrates to verify if the maximal fluxes of CAC and ketogenesis were altered in CAF livers. Octanoate, a medium chain FA, was employed because it freely reaches the mitochondrial matrix to be oxidized to ketone bodies and ^14^CO_2_ in a process not limited by carnitine-acyl transferase activity [39]. Figure 3C,D illustrate the protocol used in this perfusion experiment. From 0–10 min, the livers were perfused without exogenous substrates. Thereafter, 0.3 mM octanoate and trace amounts of [1-^14^C]octanoate (25 mCi/mmol) were simultaneously infused into the livers (10–26 min). Similar to the previous perfusion experiments, before FA infusion (basal condition), oxygen consumption tended to be lower in CAF livers, but ketogenesis and the β-hydroxybutyrate/acetoacetate ratio were not significantly different between CON and CAF livers. Both groups were rapidly sensitive to octanoate infusion and all parameters reached a new steady-state, which tended to return to basal levels upon cessation of octanoate infusion.

Comparison of AUC (Table 2) revealed that the extra ketone body production and oxygen consumption due to octanoate infusion (10–26 min) were not significantly different between both groups. However, ^14^CO_2_ production in CAF was lower than in CON livers (−24.6%, *p* < 0.05), thus reproducing the effect found with stearate plus lactate and pyruvate as substrates.

### 3.5. The Glucagon Effects on Gluconeogenesis Due to Lactate or Glycerol Infusion in Livers of Rats Fed a Standard or a Cafeteria Diet

**Figure 4:**
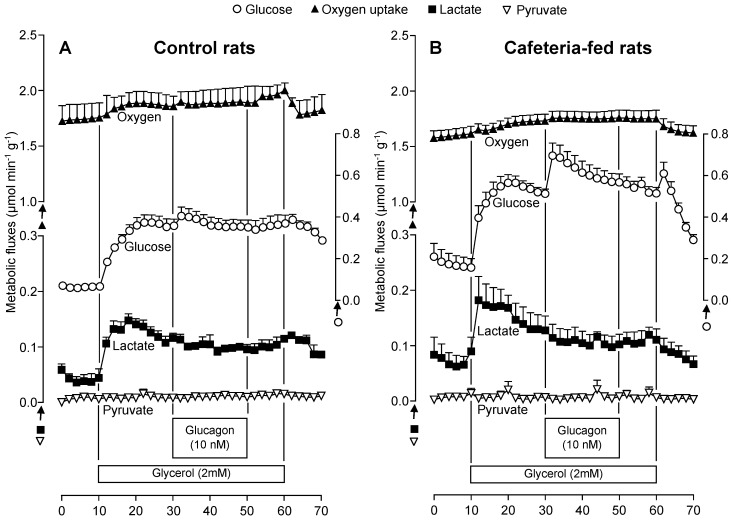

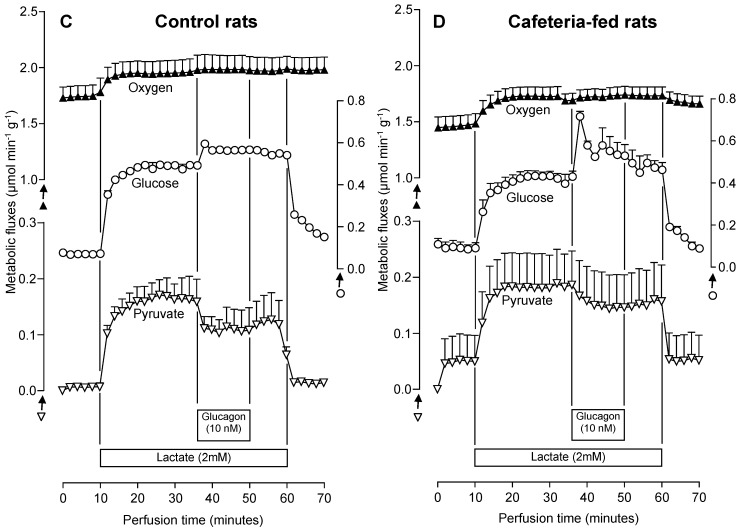
Time courses of the changes in glucose, lactate and pyruvate production and oxygen consumption due to glycerol or lactate and glucagon in perfused livers of control (**A**,**C**) and cafeteria-fed (**B**,**D**) rats. Livers of overnight (12-h) fasted rats were perfused as described in Materials and methods. Glycerol (2 mM) or lactate (2 mM) and glucagon (10 nM) were infused at the times indicated on each graph. Each experimental point is the mean ± standard error of 4–6 experiments.

To investigate whether the gluconeogenesis response to glucagon differs between fatty and normal livers, perfusion experiments were performed with lactate and glycerol as exogenous substrates, and the results are shown in Figure 4A–D. The time course of metabolic changes shows that in the absence of substrates the basal production of lactate, pyruvate, and glucose tended to be higher in CAF compared to CON livers. In contrast, the basal rates of oxygen consumption tended to be lower in CAF livers. Infusion of 2 mM glycerol or 2 mM lactate caused increment in almost all measured metabolic fluxes of both CON and CAF livers. The only exception was in pyruvate production, which was unaffected by glycerol infusion in both groups.

After the onset of 2 mM glycerol infusion, glucose production increased more rapidly in CAF compared to CON livers. At the end of 20 min, hepatic gluconeogenesis was clearly higher in CAF than in CON livers, and the infusion of glucagon further enhanced this difference. Glucose production under glucagon influence gradually decreased during the infusion period in both animal groups. Contrasting with the glycerol infusion results, in the presence of lactate, CAF livers showed a reduced glucose release rate when the steady-state was achieved. Glucagon infusion increased glucose production to a higher extent in CAF compared to CON livers, reproducing the effects found with glycerol as the substrate. The glucagon response was also relatively transitory in both groups.

The gluconeogenic substrates (glycerol or lactate) with subsequent glucagon infusion tended to increase the oxygen consumption rate in both CON and CAF livers. There was no significant difference between the groups, although the absolute values during the whole perfusion experiment period were always lower in CAF livers.

A substantial glycerol fraction metabolized by the liver was released as lactate and pyruvate (Figure 4A,B). Lactate production increased more rapidly after 2 mM glycerol infusion in CAF compared to CON livers. The steady-state values of lactate production tended to be slightly higher in CAF livers and diminished upon glucagon infusion in CON and CAF livers. Pyruvate production was very low in both groups when glycerol was infused in the absence and presence of glucagon (Figure 4A,B). When lactate (2 mM) was used as the gluconeogenic substrate, pyruvate production was similar in CON and CAF livers, but also tended to diminish in both groups upon glucagon infusion (Figure 4C,D).

The AUC values for each of these metabolic fluxes were calculated and summarized in Table 3. An insignificant difference was found between CAF and CON livers regarding oxygen consumption or metabolite production derived from endogenous substrates (basal rates). A difference between CON and CAF livers was found when the two gluconeogenic substrates were infused. Whereas glucose production was significantly higher in CAF compared to CON livers when glycerol was the substrate (+39.4%, *p* < 0.05), an opposite effect was found when lactate was infused (−21.7%, *p* < 0.05). As revealed by Table 3, for both gluconeogenic substrates, no significant difference was found between the groups regarding oxygen consumption and pyruvate and lactate production.

Confirming the alterations in metabolic flux rates due to glucagon infusion seen in Figure 4A–D, the glucagon-induced gluconeogenesis stimulation was more accentuated in CAF compared to CON livers (Table 3). The difference between CON and CAF livers was as follows: glycerol (458.5%, *p* < 0.05) and lactate (180.0%, *p* < 0.05). Glucagon tended to stimulate oxygen consumption in CON and CAF livers. The most pronounced effect of this hormone on oxygen consumption was found with lactate; CAF consumed more oxygen than CON livers (+366.7%, *p* < 0.05). With glycerol as the substrate, on the other hand, no significant difference in oxygen consumption was found between CON and CAF livers. As determined in previous conditions (basal and gluconeogenic substrates), the production of pyruvate and lactate was not significantly different between CON and CAF livers in the presence of glucagon.

### 3.6. Fatty Acid Oxidation in Isolated Mitochondria from Livers of Rats Fed a Standard or a Cafeteria Diet

The FA oxidation-driven oxygen consumption in uncoupled isolated mitochondria allows for the estimation of β-FA oxidation, citric acid cycle, and respiratory chain maximum capacity. The FAs were used as acyl-CoA derivatives (octanoyl-CoA and palmitoyl-CoA) in the presence of carnitine. Octanoyl-l-carnitine and palmitoyl-l-carnitine were also tested. As revealed by Table 4, hepatic mitochondria isolated from CAF did not exhibit significant alterations in the FA-driven respiratory rate when compared to those from CON rats.

### 3.7. [^14^C]sucrose and [^3^H]water Outflow Profiles and Related Parameters in Livers of Rats Fed a Standard or a Cafeteria Diet

**Figure 5:**
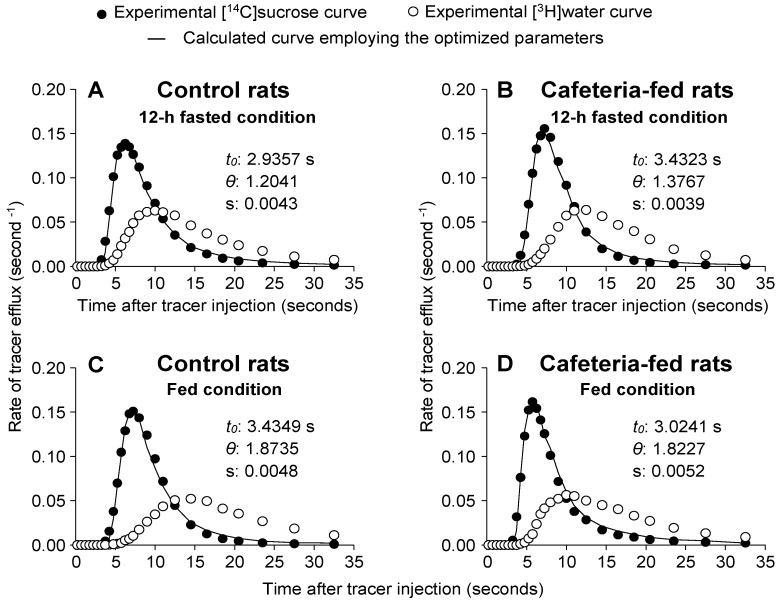
Influence of cafeteria diet-induced obesity on typical outflow profiles of [^14^C]sucrose and [^3^H]water. As indicated in the graphs, livers of overnight (12-h) fasted (**A**,**B**) or fed (**C**,**D**) rats were perfused in an open system with Krebs/Henseleit-bicarbonate buffer (pH 7.4) according to the procedure described in Materials and methods. Fractions of the injected radioactivity of each component appearing in the effluent perfusate per second are represented against the time after injection. The continuous line represents the optimized superposition of the [^3^H]water on the [^14^C]sucrose curve according to Equation (3). The optimized values of *t*_0_ and *θ* were obtained from a linear superposition of the [^3^H]water and [^14^C]sucrose curves (according to Equation (3)). The letter (s) represents the standard error of the estimate. The curves in panels A, B, C and D are representative of 5–6 indicator-dilution experiments.

To evaluate whether the metabolic changes found in CAF livers, especially alterations in oxygen consumption, could be a consequence of disturbances in distribution spaces, MID experiments were performed in the following four experimental conditions: (1) overnight (12 h) fasted CON rats, (2) overnight (12 h) fasted CAF rats, (3) fed CON rats, or (4) fed CAF rats. Figure 5A–D shows the typical outflow profiles of experiments in which [^3^H]water and [^14^C]sucrose were injected into the livers. All curves were normalized by dividing the radioactivity amount that reappeared per second by the total injected radioactivity. If there was no loss or sequestration of [^3^H]water or [^14^C]sucrose within the liver, the areas under such normalized curves were equal to unity, as happened with all curves obtained in the present study. As expected, in all curves the [^3^H]water outflow profile was delayed in relation to the [^14^C]sucrose outflow profile because water distributes over the entire aqueous space of the liver, whereas [^14^C]sucrose does not exchange with the cellular space during a single passage.

Quantitative parameters of distribution spaces can be obtained from experimental curves of [^3^H]water and [^14^C]sucrose, including the mean transit time of tracers $\left(\overset{¯}{t}\right)$, the transit time in the large vessels (*t*_0_), and the ratio of intracellular to extracellular water spaces (*θ*). The parameters *t_0_* and *θ* were derived from an optimized superposition of the normalized [^3^H]water (*Q_water_*(*t*)) outflow profile on the [^14^C]sucrose (*Q_suc_*(*t*)) outflow profile, according to Equation (3) [33]. Figure 5A–D reveals good agreement between theory (continuous line) and experiment (closed circles). The optimized parameters (*t*_0_ and *θ*) and standard errors of the estimates are also shown in Figure 5A–D. The quantitative data illustrated in Figure 5A–D is listed in Table 5. There was no significant difference between all groups with respect to *t*_0_. The *θ* values were higher in fed rats compared to 12 h fasted rat livers, regardless of the diet (CON or CAF diet). The *θ* values of fed CON and CAF livers were higher by 52.5% and 56.7%, respectively, compared to their fasted counterparts (*p* < 0.05). These higher *θ* values in fed groups agreed with the significantly higher ${\overset{¯}{t}}_{water}$ (tritiated water mean transit time), with no change in ${\overset{¯}{t}}_{sucrose}$ (labelled sucrose mean transit time). The ${\overset{¯}{t}}_{water}$ of CON and CAF livers (in fed condition) were higher by 17.3% and 27.3%, respectively, compared to the same groups in the fasting condition (*p* < 0.05). In this case, ${\overset{¯}{t}}_{water}$ tended to be lower in CAF compared to CON livers (*p* = 0.072).

The parameters obtained from the linear superimposition (Equation (3)) and the mean transit times (Equation (4)) allowed for the calculation of the liver distribution spaces by multiplying the flow rate through the perfused liver (volume/time) with specific combinations of these parameters [33,35]. The V_e_ (sinusoidal extracellular space accessible to labelled sucrose, i.e., sinusoid + Disse space) can be calculated as flow × $\left({\overset{¯}{t}}_{sucrose}-t_{0}\right)$. The V_i_ (cellular aqueous space of the liver) can be determined as flow × $\left({\overset{¯}{t}}_{water}-{\overset{¯}{t}}_{sucrose}\right)$ [40]. In the overnight (12 h) fasted condition, V_e_ tended to be lower in CAF compared to CON livers. This difference was significant in the fed condition so that V_e_ was 24.0% lower in CAF compared to CON livers (*p* < 0.05). The fed showed a lower V_e_ (−32.1%, *p* < 0.05) than the fasted CAF animals. V_i_ was 13.2% (*p* < 0.05) lower in CAF compared to CON livers in the fasted condition. A similar change was found in the fed condition (−18.2%, *p* < 0.05). The V_i_ of fed CON livers, however, was 15.8% higher (*p* < 0.05) when compared to the same fasted group. The V_i_ to V_e_ ratio was not significantly different in both groups, independent of the condition. This ratio was higher in fed compared to overnight (12 h) fasted animals. The increase was +50.8% and +54.2%, respectively, for CON and CAF groups (*p* < 0.05). Table 5 shows that the large non-exchanging vessel (V_v_) volume, calculated as flow × *t*_0_, was not different in CAF compared to CON livers in fasting. In the fed condition, CAF presented lower V_v_ values (−27.3%, *p* < 0.05) than those of CON livers. The CAF livers of fed rats also presented lower V_v_ values (−33.3%, *p* < 0.05) than those of fasted CAF rat livers.

## 4. Discussion

The present study demonstrated that the cafeteria diet administration for 14 weeks in weaned rats induced high body weight gain, increased adiposity, increased liver wet weight, high hepatic glycogen levels, and hepatic steatosis in the adult phase. These alterations are in accordance with previous studies using a cafeteria diet-induced obesity experimental model [4,17,22,23]. Curiously, despite the augmented energy intake of a cafeteria diet, expressive body weight gain appeared only after the 9th week, suggesting a pre-existing resistance to develop obesity at an early age. This behaviour has been previously exhibited in mice and rats [22,41,42]. The high lipid and glycogen content in the liver, as well as the increased liver wet weight, clearly represents the adaptive metabolic changes to a new nutritional condition due to a cafeteria diet.

**Figure 6:**
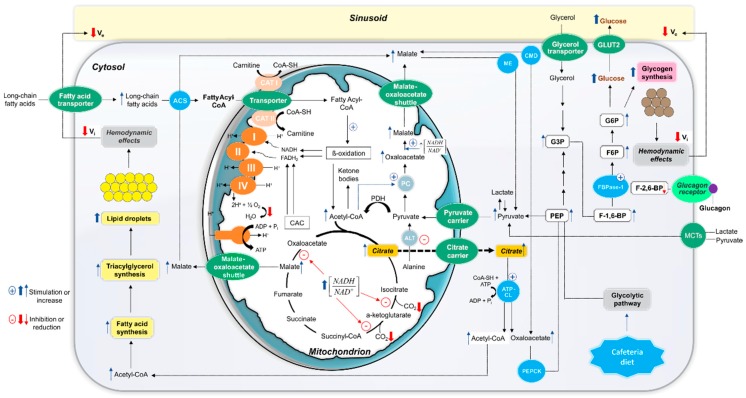
Schematic representation of important events that likely occur in vivo, leading to augmented gluconeogenesis in the presence of fatty acids and glucagon, enhanced glycogen synthesis and hemodynamic alterations in the steatotic liver cells of cafeteria-diet-induced obese rats. The scheme is debated in the text and is based on results of the present and earlier published works. The thick arrows indicate the experimentally observed changes and the thin arrows the changes that likely occurred. The upward arrows and (+) symbol denote the increased enzyme activity or metabolite concentration, whereas the downward arrows and (−) symbol the reduced enzyme activity or metabolite concentration. Abbreviations: ACS, fatty acyl-CoA synthetase; CAT, carnitine acyltransferase; ALT, alanine aminotransferase; CAC, citric acid cycle; PDH, pyruvate dehydrogenase complex; ME, malic enzyme; CMD, cytosolic malate dehydrogenase; ATP-CL, ATP citrate lyase; PEPCK, phosphoenolpyruvate carboxykinase; PEP, phosphoenolpyruvate; G3P, glyceraldehyde 3-phosphate; F-1,6-BP, fructose 1,6-bisphosphate; F-2,6-BP, fructose 2,6-bisphosphate; FBPase-1, fructose 1,6-bisphosphatase; F6P, fructose 6-phosphate; G6P, glucose 6-phosphate; GLUT2, glucose transporter 2; MCTs, monocarboxylate transporters; V_i_, accessible cellular space; V_e_, sinusoidal + Disse space.

In this study, we have focused on a possible positive modulation role of free fatty acids (FFA) and glucagon on gluconeogenesis in NAFLD rat livers. A reduced gluconeogenesis rate was found in CAF livers when lactate + pyruvate and lactate were the substrates, corroborating our previous study with l-amino acids as substrates [17], but, in contrast, an increase was observed when glycerol was the substrate. Despite the lower gluconeogenic rates from lactate + pyruvate, the concomitant presence of long-chain FA stearate caused a higher stimulation in CAF livers. A similar phenomenon was observed regarding glucagon, which caused a higher stimulatory effect on gluconeogenesis in CAF livers, irrespective of the fact that gluconeogenesis was lower from lactate and higher from glycerol in these livers compared to the CON condition. These findings clearly indicate that the observed changes in hepatic glucose production are a consequence of a complex pattern of interaction between pathways specifically involved in the metabolism of gluconeogenic substrates and effectors (lactate, pyruvate, glycerol, and stearate) and hormonal regulatory systems. The main aspects involved in the altered metabolic behaviour of CAF livers are summarized in Figure 6. One must consider not only the glucose production rate from a specific substrate, but also the changes in other related metabolic and physical aspects. It is possible to devise at least the following four additional potential factors influencing the gluconeogenic capacity of CAF livers: (a) changes in the cellular NADH/NAD^+^ redox couple, (b) alterations in activities of enzymes catalysing gluconeogenesis key steps and their hormonal regulation, (c) particular conditions generated by FA metabolism, and (d) hepatic hemodynamic changes.

Gluconeogenesis from lactate and pyruvate occurs under an adequate NADH supply, essential for the glyceraldehyde 3-phosphate dehydrogenase step [43]. The gluconeogenic first step from lactate consists of its oxidation to pyruvate catalysed by lactate dehydrogenase, generating cytosolic NADH. In the case of glycerol, however, NAD^+^ is required to convert glycerol 3-phosphate into dihydroxyacetone phosphate and glyceraldehyde 3-phosphate. The findings that CAF liver gluconeogenesis was depressed from lactate and pyruvate whereas CAF liver gluconeogenesis increased from glycerol were not consistent with changes in the cytosolic NADH/NAD^+^ ratio as a factor directly involved in gluconeogenesis alteration. A disturbance in the mitochondrial redox state, however, seems to have an important influence. In CAF livers, an increased mitochondrial NADH/NAD^+^ ratio was found, as indicated by the increased β-hydroxybutyrate to acetoacetate ratio observed in the presence of gluconeogenic substrates (e.g., lactate and pyruvate), principally in the presence of stearic acid. Whenever the mitochondrial matrix redox environment is under a highly-reduced state, CAC inhibition is an expected response [44,45]. That this happened in CAF livers was supported by the reduced ^14^CO_2_ production when [^14^C]stearic acid was infused with lactate and pyruvate and when [^14^C]octanoic acid was infused in the absence of gluconeogenic substrates. This reduction was not related to a mitochondrial respiratory chain impairment since oxygen consumption due to FA oxidation was not altered in isolated mitochondria. Under this condition, citrate produced from oxaloacetate and acetyl-CoA condensation in the CAC first step probably accumulates in the mitochondrial matrix, as isocitrate-dehydrogenase is one of the key cycle rate-limiting steps regulated by the NADH/NAD^+^ ratio [46]. Citrate can thus be exported to the cytosol via a citrate carrier, where it is converted to acetyl-CoA and oxaloacetate by ATP-citrate lyase. These events finally result in the draining of mitochondrial oxaloacetate, an essential metabolite for the first step of lactate and pyruvate gluconeogenesis [47]. This hypothesis is consistent not only with reduced glucose production when lactate and pyruvate were the substrates, but also with reduced ketone body production from endogenous sources. CAC inhibition at the citrate synthase step was unlikely since this effect would result in acetyl-CoA accumulation and its diversion to ketone body production [48].

Supporting our hypothesis, it has been reported that elevated citrate levels are one of the primary causes of hepatic steatosis development in humans and rodent models of obesity [46,49,50]. It is believed that the energy excess within the liver cells, due to high glucose and free FA levels, leads to both increased acetyl-CoA generation and mitochondrial NADH/NAD^+^ ratio, favouring citrate formation and subsequent export [46]. As citrate is a powerful acetyl-CoA carboxylase inducer in cytosol, the malonyl-CoA accumulation suppresses FA oxidation and gluconeogenesis from lactate and pyruvate, effects that were indeed observed in CAF livers. Also, in agreement with this interpretation, gluconeogenesis from glycerol was not reduced in CAF livers, but, in contrast, an increase was found. Results from our previous study with alanine and glutamine as gluconeogenic substrates [17] indicated that the cytosolic enzymes for the phosphoenolpyruvate (PEP) conversion into glucose are not impaired in CAF livers. This interpretation is also supported by the observation that glucose production was further increased when stearate or glucagon were infused. With lactate and pyruvate as substrates, the concomitant stearate infusion caused a 4.4-fold stimulation in glucose production, despite the lower rates in its absence. This strong stimulatory effect of FFA on gluconeogenesis in NAFLD rat livers had not been described so far. Taken together, our results allow a probable sequence of events to explain this effect (Figure 6).

The exogenous stearate infusion added additional fuel to support the energy cost of gluconeogenesis due to activation of mitochondrial FA β-oxidation. Also, the lower CAC activity indicated by reduced CO_2_ production from [^14^C]stearic acid and [^14^C]octanoic acid probably led to extra mitochondrial acetyl-CoA production and shifted the allosteric regulation of pyruvate carboxylase towards activation since acetyl-CoA is a potent stimulator of this enzyme [51,52,53]. In addition, the higher citrate exported into the cytosol in CAF livers, as discussed above, would result in higher oxaloacetate levels derived from the cytosolic ATP-citrate lyase action, favouring PEP carboxykinase activity with formation of PEP. Moreover, higher FA oxidation due to stearate infusion would lead to a higher mitochondrial NADH/NAD^+^ ratio, as indicated by the enhanced β-hydroxybutyrate to acetoacetate ratio. Under this condition, mitochondrial oxaloacetate export (as malate) could be further increased due to mitochondrial l-malate dehydrogenase stimulation [54,55]. Therefore, the increased PEP cytosolic levels also seem to play an important role in the stearate-induced gluconeogenesis activation in CAF livers. It should also be considered that exogenous stearate metabolism could be favoured by the increased uptake in CAF livers since upregulation of the long-chain FA transport proteins, mainly FATP2 and FATP5, has been reported in other models of experimental obesity and steatosis [56,57,58,59].

The glucagon action is probably linked to regulation of key enzymes related to the following fructose-1,6-biphosphate steps since its higher stimulating action was observed in CAF livers, irrespective of the exogenously gluconeogenic substrate (glycerol or lactate). It is well known that the glucagon regulatory mechanism in gluconeogenesis consists of lowering intracellular levels of fructose-2,6-bisphosphate, a potent inactivator of fructose-1,6-biphosphatase [60]. Glycerol comes in the gluconeogenic pathway as a triose-phosphate and its conversion into glucose involves fructose-1,6-biphosphatase. Transformation of lactate and pyruvate, on the other hand, depends on the flux through the pyruvate/PEP cycle and fructose-1,6-biphosphatase activity. Thus, it is likely that there is a higher activation of this enzyme by glucagon signalling in CAF livers. This interpretation is corroborated by reports of glucose overproduction in obese animal models, associated with increased fructose-1,6-biphosphatase activity and protein levels [61,62]. Our results are also in line with previous findings [63] in which the glucagon stimulating action in gluconeogenesis is more pronounced at a high NADH/NAD^+^ ratio. It should be mentioned that elevated plasma levels of this hormone have been found in obese, prediabetic, and type 2 diabetic conditions [20,64,65]. In the isolated perfused rat liver, the gluconeogenesis rate relies on the liver enzymatic capacity, which reflects the medium- and long-term effects of the circulating hormones (e.g., insulin/glucagon ratio in the portal vein) [66]. Therefore, an upregulation of genes coding for gluconeogenic enzymes such as fructose-1,6-biphosphatase is a real possibility in CAF rat livers.

Once glucagon and FFA plasma levels are frequently elevated in the NAFLD, an increased hepatic gluconeogenesis rate in the presence of FAs and glucagon is a phenomenon that probably predominates under in vivo conditions in CAF rats, favouring the increased glycogen content found in these animals in both the fed and fasted condition. It is known that in the fed state gluconeogenesis contributes to hepatic glycogen formation by nearly 23%; however, this proportion can reach 65% in fasting [67]. As demonstrated in our previous study [17], plasma glucose and triglyceride concentrations, which are classical risk factors of insulin resistance [68], are elevated in this model of cafeteria diet-induced obesity. Other authors also found that this animal model of obesity is associated with insulin resistance [69,70,71]. Under the hyperinsulinemic condition, liver glycogen synthesis is activated through a marked increase in the indirect pathway (glucose from gluconeogenesis). This, in turn, may compensate for reduction in the direct pathway (from exogenous glucose) once hepatic fat accumulation decreases insulin activation of glycogen synthase [72,73]. A probable mechanism underlying the indirect pathway of glycogen synthesis in CAF livers is an increased supply of gluconeogenic precursors, e.g., glycerol [72]. Although the plasma concentration of glycerol was not measured in the present study, other investigators have demonstrated that in obese and insulin resistant animals, glycerol concentration is high in the portal vein [74,75]. This data thus reinforces our hypothesis that the high gluconeogenesis rate from glycerol can increase glycogen content and hyperglycemia.

The results of MID experiments revealed that the high glycogen and lipid content in hepatocytes induced modifications in liver distribution spaces. In fasted CAF rat livers, reduction in accessible cellular water space was found, probably as a consequence of [^3^H]water exclusion by excess lipid droplets. It seems that these inclusions caused a ballooning of hepatocytes and, consequently, reduction in extracellular spaces, as indicated by reductions in V_e_ (sinusoid + Disse space) as well as in the large non-exchanging vessel volume (V_v_). This phenomenon was more evident in fed compared to fasted rat livers, possibly due to the higher glycogen accumulation in addition to lipid droplets. These hemodynamic changes could explain the reduced oxygen consumption rates in CAF livers, even in the presence of exogenously added substrates. A direct alteration in enzymes of the mitochondrial respiratory electron chain was unlikely to contribute to this reduction because no difference was found in the maximal respiration capacity during FA oxidation in isolated mitochondria. Reductions in vascular and extravascular volumes can lead to the exclusion of a liver parenchyma fraction from the microcirculation, thus restricting the access of oxygen [76,77,78,79]. This exclusion does not seem to reduce the energy supply to gluconeogenesis since no limitation was observed in this pathway. These findings are in accordance with previous reports [80], which showed that the reductions in gluconeogenesis attributable to decreases in hepatic blood flow are primarily due to compromised precursor delivery, not oxygen availability. Additionally, because gluconeogenesis is known to predominate in hepatocytes located in the periportal hepatic parenchyma [81,82,83], richer in oxygen than the perivenous zone, it is likely a restriction in oxygen supply due to hemodynamic changes mainly affected hepatocytes in the perivenous area and the restriction in oxygen supply suppresses FA oxidation and enhances lipid accumulation in hepatocytes [84], which could further aggravate the progression of NAFLD.

In summary, our data reveals new findings on the consequences of feeding rats at early stages of life with a cafeteria diet on hepatic gluconeogenesis and its possible roles in hyperglycemia in the adult phase. We have found that gluconeogenesis from substrates dependent on enzymatic steps before PEP generation is reduced, including glucose production from lactate and pyruvate. Gluconeogenesis from glycerol, however, is stimulated in these rats. Moreover, in the presence of FAs or glucagon, e.g., in the in vivo condition, gluconeogenesis stimulation is higher in CAF compared to CON rat livers, indicating that the gluconeogenic capacity of steatotic livers is not impaired. The higher gluconeogenesis rates probably contribute to hyperglycemia and higher glycogen deposition in livers of adult rats. The greater content of glycogen granules and lipid droplets seem to trigger hepatic hemodynamic changes, reducing the cellular and extracellular volumes, thus restricting the oxygen supply in liver parenchyma. Our results, along with data published in previous studies, allowed us to propose the mechanistic basis for these assumptions, as discussed above and illustrated in Figure 6. It can be concluded that, although hepatic insulin resistance has been suggested as the main factor inducing enhanced gluconeogenesis and hyperglycemia in the obesity condition, our study indirectly revealed that high levels of circulating free FAs and glucagon also exert an important influence. Moreover, we have provided evidence that cafeteria diet feeding at early stages of life can change the liver to be overresponsive to the influence of FAs and glucagon on gluconeogenesis during the adult phase in rats. These findings may provide evidence for the discovery of potential targets for antihyperglycemic therapies.

## Figures and Tables

**Table 1:** Weight-related parameters of cafeteria-fed and control rats.

Parameters	Control Rats	Cafeteria-Fed Rats
Initial body weight (g)	50.70 ± 2.00 (*n* = 21)	51.10 ± 2.60 (*n* = 21)
Final body weight (g)	416.00 ± 4.50 (*n* = 21)	485.00 ± 6.60 * (*n* = 21)
Body weight gain (g)	365.00 ± 4.40 (*n* = 21)	434.00 ± 8.20 * (*n* = 21)
Adiposity index (g × 100 g of body weight^−1^)	4.45 ± 0.28 (*n* = 10)	8.96 ± 0.49 * (*n* = 10)
Liver wet weight (g)	11.90 ± 0.37 (*n* = 10)	14.05 ± 0.50 * (*n* = 10)
Hepatic glycogen in fed rats (µmol glucosyl units × g^−1^)	139.10 ± 18.90 (*n* = 7)	256.00 ± 14.90 * (*n* = 4)
Hepatic glycogen in 12-h fasted rats (µmol glucosyl units × g^−1^)	6.69 ± 1.72 (*n* = 10)	14.30 ± 1.45 * (*n* = 9)
Hepatic total lipid content (mg × g liver wet weight^−1^)	19.16 ± 0.77 (*n* = 4)	58.72 ± 7.82 * (*n* = 4)
Hepatic total protein content (mg × g liver wet weight^−1^)	177.50 ± 6.33 (*n* = 4)	173.50 ± 4.39 (*n* = 4)

Except for the measurement of initial body weight of rats, all other parameters were measured at the end of treatment (14 weeks). The values are expressed as the means ± standard error of the means of 4–21 experiments, depending on the occasion. The adiposity index was calculated from the sum of the retroperitoneal, mesenteric, periepididymal and interscapular fat weights, which was related to g/100 g of body weight. * *p* < 0.05 as compared with the control group, according to Student’s *t*-test.

**Table 2:** Metabolic parameters related to gluconeogenesis and fatty acid oxidation in perfused livers from control and cafeteria-fed rats.

Parameter	[Lactate] + [Pyruvate] (mM)	[Fatty Acid] (mM)	Δ Metabolite Production (μmol × g^−1^)	
			Control Rats	Cafeteria-Fed Rats
Glucose	0	0	0.98 ± 0.05 (*n* = 4)	2.18 ± 0.56 (*n* = 4)
	2.0 + 0.2	0	7.84 ± 0.82 (*n* = 4)	4.58 ± 0.40 * (*n* = 4)
	2.0 + 0.2	0.2 Stearate	0.51 ± 0.10 (*n* = 4)	2.25 ± 0.70 * (*n* = 4)
Ketone bodies	0	0	4.84 ± 0.19 (*n* = 8)	4.04 ± 0.21 * (*n* = 9)
	2.0 + 0.2	0	−6.25 ± 0.23 (*n* = 4)	−4.72 ± 0.05 * (*n* = 4)
	2.0 + 0.2	0.2 Stearate	0.64 ± 0.19 (*n* = 4)	0.56 ± 0.10 (*n* = 4)
	0	0.3 Octanoate	8.49 ± 0.90 (*n* = 4)	7.26 ± 1.20 (*n* = 5)
O_2_ consumption	0	0	18.74 ± 0.66 (*n* = 8)	15.30 ± 0.34 * (*n* = 9)
	2.0 + 0.2	0	4.78 ± 0.37 (*n* = 4)	4.38 ± 0.81 (*n* = 4)
	2.0 + 0.2	0.2 Stearate	0.70 ± 0.15 (*n* = 4)	0.84 ± 0.25 (*n* = 4)
	0	0.3 Octanoate	4.23 ± 0.81 (*n* = 4)	3.57 ± 0.35 (*n* = 5)
CO_2_	2.0 + 0.2	0.2 Stearate	14.22 ± 1.02 (*n* = 4)	10.70 ± 0.60 * (*n* = 4)
	0	0.3 Octanoate	6.30 ± 0.54 (*n* = 4)	4.75 ± 0.40 * (*n* = 5)

The data were obtained from the experiments shown in Figure 3 and represent the means ± standard errors of the means of the areas under the metabolite production versus time curves. Calculations were made as described in the Results section. Values are expressed as means ± standard errors of the means (*n* = number of experiments) and represent the area under metabolite production versus time curves during the infusion of no substrate (first 10 min of perfusion time in Figure 3A–D), during the infusion of 2 mM lactate + 0.2 mM pyruvate (10–30 min of perfusion time in Figure 3A,B), during the infusion of 0.2 mM stearate in the presence of 2 mM lactate + 0.2 mM pyruvate (30–46 min of perfusion time in Figure 3A,B) and in the presence of 0.3 mM octanoate in the absence of gluconeogenic substrates (10–26 min of perfusion time in Figure 3C,D). Negative values represent decreases in production of metabolites. * *p* < 0.05 as compared with the control group, according to Student’s *t*-test.

**Table 3:** Metabolic parameters related to gluconeogenesis due to lactate or glycerol and glucagon in perfused livers from control and cafeteria-fed rats.

**Conditions**	**Δ Metabolite Production (μmol × g^−1^)**							
	**Control Rats**				**Cafeteria-Fed Rats**			
**Glycerol Gluconeogenesis**	**Glicose**	**O_2_ Consumption**	**Lactate**	**Pyruvate**	**Glicose**	**O_2_ Consumption**	**Lactate**	**Pyruvate**
Basal production	0.64 ± 0.09	17.41 ± 1.37	0.41 ± 0.10	—	1.74 ± 0.51	15.96 ± 0.62	0.71 ± 0.20	—
Glycerol (2 mM)	4.75 ± 0.65	1.97 ± 0.56	1.54 ± 0.38	—	6.62 ± 0.45 *	1.48 ± 0.46	1.25 ± 0.35	—
Glycerol (2 mM) + Glucagon (10 nM)	0.41 ± 0.13	0.86 ± 0.50	−0.29 ± 0.04	—	2.29 ± 0.50 *	0.39 ± 0.10	−0.42 ± 0.12	—
**Conditions**	**Δ Metabolite Production (μmol × g^−1^)**							
	**Control Rats**				**Cafeteria-Fed Rats**			
**Lactate Gluconeogenesis**	**Glicose**	**O_2_ Consumption**	**Lactate**	**Pyruvate**	**Glicose**	**O_2_ Consumption**	**Lactate**	**Pyruvate**
Basal production	0.70 ± 0.03	17.45 ± 1.00	—	0.05 ± 0.04	0.92 ± 0.27	14.62 ± 0.92	—	0.44 ± 0.41
Lactate (2 mM)	9.59 ± 0.17	3.97 ± 0.46	—	3.55 ± 0.68	7.51 ± 0.15 *	5.34 ± 0.61	—	3.06 ± 0.42
Lactate (2 mM) + Glucagon (10 nM)	0.90 ± 0.07	0.09 ± 0.03	—	−0.60 ± 0.11	1.62 ± 0.25 *	0.42 ± 0.11 *	—	−0.42 ± 0.07

The data were obtained from the experiments shown in Figure 4 and represent the means ± standard errors of the areas under the metabolite production versus time curves. Calculations were made as described in the Results section. Values are expressed as means ± standard errors of the means of 4–6 perfusion experiments and represent the area under metabolite production versus time curves during infusion of no substrate (first 10 min of perfusion time in Figure 4A–D), during the infusion of 2 mM glycerol (1–30 min of perfusion time in Figure 4A,B), during the infusion of 2 mM lactate (10–36 min of perfusion time in Figure 4C,D) and during the infusion of 10 nM glucagon in the presence of 2 mM glycerol (30–50 min of perfusion time in Figure 4A,B) or in the presence of 2 mM lactate (36–50 min of perfusion time in Figure 4C,D). Negative values represent decreases in production of metabolites. * *p* < 0.05 as compared with the control group, according to Student’s *t*-test.

**Table 4:** The influence of cafeteria diet-induced obesity on the oxygen consumption by mitochondria performing fatty acid oxidation.

Group (n)	Octanoyl-CoA + l-Carnitine	Palmitoyl-CoA + l-Carnitine	Octanoyl l-Carnitine	Palmitoyl l-Carnitine
Control rats (6)	40.5 ± 4.3	59.0 ± 3.3	61.3 ± 2.6	77.0 ± 5.9
Cafeteria-fed rats (6)	37.0 ± 3.2	64.5 ± 3.1	63.5 ± 3.9	88.5 ± 5.8

Liver mitochondrial fatty acid β-oxidation was measured by polarography in the presence of 0.1 mM 2,4-dinitrofenol. Reactions were initiated by the addition of the following: 20 µM palmitoyl-l-carnitine, 20 µM palmitoyl-CoA + 2.0 mM l-carnitine, 20 µM octanoyl-l-carnitine or 20 µM octanoyl-CoA + 2.0 mM l-carnitine. Mitochondria (1.0 mg protein/mL) were incubated in final volumes of 2.0 mL. Values are expressed in nmol O_2_ × min^−1^ × mg protein^−1^ as means ± standard error of the means. (n) = number of experiments. No differences were found between groups in any measured respiratory variable according to Student’s *t*-test.

**Table 5:** Transit times, cellular spaces and vascular spaces in the isolated perfused rat liver of control and cafeteria diet-induced obese rats.

Parameter	12-h Fasted Rats		Fed Rats	
	Control Rats (*n* = 6)	Cafeteria-Fed Rats (*n* = 6)	Control Rats (*n* = 5)	Cafeteria-Fed Rats (*n* = 5)
*t*_0_ (seconds)	3.06 ± 0.29	2.99 ± 0.21	3.08 ± 0.29	2.91 ± 0.12
*θ*	1.18 ± 0.07 *	1.20 ± 0.06 ^#^	1.80 ± 0.12	1.88 ± 0.10
${\overset{¯}{t}}_{sucrose}$ (seconds)	10.48 ± 0.58	9.73 ± 0.35	10.14 ± 0.49	9.78 ± 0.16
${\overset{¯}{t}}_{water}$ (seconds)	19.24 ± 0.70 *	17.64 ± 0.38 ^#^	22.57 ± 0.55	22.47 ± 0.81
V_e_ as flow × $\left({\overset{¯}{t}}_{sucrose}-t_{0}\right)$ (mL/g)	0.33 ± 0.03	0.28 ± 0.02 ^#^	0.25 ± 0.02	0.19 ± 0.01 °
V_i_ as flow × $\left({\overset{¯}{t}}_{water}-{\overset{¯}{t}}_{sucrose}\right)$ (mL/g)	0.38 ± 0.01 *	0.33 ± 0.01 ^∆^	0.44 ± 0.02	0.36 ± 0.02
V_i_/V_e_	1.20 ± 0.08 *	1.20 ± 0.08 ^#^	1.81 ± 0.11	1.85 ± 0.08
V_v_ as flow × *t*_0_ (mL/g)	0.14 ± 0.02	0.12 ± 0.01 ^#^	0.11 ± 0.01	0.08 ± 0.00 °

The multiple indicator dilution experiments were performed as described in Materials and methods section. The *t*_0_ and *θ* values were obtained from the superposition of the [^14^C]sucrose and [^3^H]water curves, according to Equation (3). The values of *t*_0_, ${\overset{¯}{t}}_{sucrose}$ and ${\overset{¯}{t}}_{water}$ were corrected for the delay produced by the collecting system. In the equations that were used for the calculation of V_e_ (sinusoidal extracellular space accessible to labeled sucrose) and V_i_ (the cellular aqueous space of the liver) the flow was expressed as mL × second^−1^ × (g liver wet weight)^−1^. V_v_ represents the volume of the large non-exchanging vessels. All parameters were obtained from indicator dilution experiments of the kind illustrated by Figure 5. Data are means ± standard errors of the means of 5 to 6 experiments. The symbols indicate the statistical significance as revealed by Student’s *t*-test (*p* < 0.05). * 12-h fasted control rats vs. fed control rats; ^#^ 12-h fasted cafeteria rats vs. fed cafeteria rats; ° fed control rats vs. fed cafeteria rats; ^∆^ 12-h fasted control rats vs. 12-h fasted cafeteria rats.
